# Ocular steroidome in human eyes and in eyes with complex central serous chorioretinopathy (CSCR)

**DOI:** 10.1038/s41598-023-41126-0

**Published:** 2023-08-29

**Authors:** Marta Zola, Elodie Bousquet, Jean-Louis Bourges, Fréderic Azan, Min Zhao, Thara Jaworski, Eric Pussard, Francine Behar-Cohen

**Affiliations:** 1 Department of Ophthalmology, Cochin Hospital, Assistance Publique-Hôpitaux de Paris, University of Paris Cité, Paris, France; 2 Centre de Recherche des Cordeliers, INSERM, Physiopathology of Ocular Diseases: Therapeutic Innovations, University Paris Cité, Paris, France; 3 Department of Ophthalmology, Hôpital Foch, Suresnes, France; 4 Department of Genetic and Hormonology, Bicêtre Hospital, Assistance Publique-Hôpitaux de Paris, University of Paris-Saclay, Le Kremlin Bicêtre, France

**Keywords:** Metabolism, Neurophysiology, Biomarkers, Diseases, Endocrinology, Medical research, Molecular medicine

## Abstract

The exact link between systemic and ocular endogenous corticoids (steroidome) is unclear and whether the ocular steroidome is altered in CSCR eyes is unknown. The aims of this study were to analyze the human steroidome in the aqueous humor as a function of age, sex and time of the day, to correlate systemic and ocular steroidome and to analyze the ocular steroidome in long lasting complex inactive CSCR. Based on our results, we present two CSCR cases treated by the combination of oral mineralocorticoid antagonist and glucocorticoids drops. In a cross-sectional study, aqueous humor (AH) was collected between 8am and 6 pm from 50 unaffected individuals (25 men and 25 women) and from 14 patients with chronic CSCR, during cataract surgery. In addition, simultaneous serum and AH were collected from 27 individuals undergoing cataract surgery and, simultaneous AH and vitreous were collected from 9 patients undergoing cataract and vitrectomy to estimate corticoids levels in the different compartments. The steroidome was determined using a LC–MS/MS method that quantifies 13 endogenous corticoids from the gluco, mineralocorticoid and androgen pathways. In AH and vitreous, the highest corticoid level is reached by cortisol (F), that represents less than 10% of F serum level. The cortisol levels in the serum did not correlate with ocular cortisol levels. Serum and ocular cortisone (E) levels correlate, although less than 5% of circulating E reaches the eye. The only mineralocorticoids measured in the AH were corticosterone (B) and its inactive form, the 11-desoxycorticosterone (A). There was no influence of circadian rhythm on cortisol ocular levels and there was no correlation between the age or the sex and the level of F, E, A, and B. In eyes with chronic inactive CSCR, the levels of the active glucocorticoid form F was lower than in control eyes and the F/E ratio was reduced by 50% but the B/A ratio was higher indicating imbalance towards active mineralocorticoids. Base on this observation, we propose to combine an antagonist of the mineralocorticoid receptor together with topical glucocorticoids in two CSCR patients, resistant to all other treatments, with favorable outcome. Our results indicate that the ocular psteroidome is highly regulated suggesting a local metabolism of ocular corticoids. In eyes with long-lasting complex inactive CSCR, the steroidome analysis shows lower active glucocorticoids and higher active mineralocorticoids.

## Introduction

Central serous chorioretinopathy (CSCR) is a chorioretinal disease belonging to the spectrum of pachychoroid, a choroid phenotype characterized by vasodilatation of the choroidal veins, indicating alteration of the hemodynamic regulation of the choroid. Within this spectrum, CSCR is characterized by the occurrence of fluctuating serous detachments of the neuroretina secondary to focal ruptures of the barrier formed by the retinal pigmentary epithelium (RPE). In about 30% of CSCR cases, recurrences, chronicity, and especially the underlying RPE degeneration and the neovascular complications can lead to permanent visual loss^1–3^.The presence of extended RPE alteration suggest long-lasting and complex disease^4^. CSCR mainly affects middle-aged men but can also occur in women at an older age^5^. The mechanisms of the disease remain imperfectly understood, involving venous overload^6^, anatomical^7^, genetic^8, 9^ and various risk factors^10, 11^.

Among the known risk factors, exogenous glucocorticoids intake is unanimously recognized. It is thus particularly interesting to better understand the mechanisms through which corticoids could act on CSCR. The risk of CSCR is higher amongst patients who have used glucocorticoids (GC), administered either orally, through non ocular injections or through nasal spray, even several weeks or months before the episode^12, 13^. But, CSCR is not favored by the intraocular administration of GCs although high levels of glucocorticoids were measured in a sustained manner in all the ocular tissues after Ozurdex (dexamethasone) or Illuvien (fluocinolone acetonide) implantation^14, 15^, indicating that high dose of GCs in RPE and/or choroid might not be sufficient to cause CSCR. The fact that the risk of CSCR differs according to whether glucocorticoids are administered extra- or intraocularly suggests that braking of the hypothalamus-pituitary axis (HPA), and hence the functioning of the adrenal gland and the metabolism of endogenous corticoids may be involved. Indeed, intraocular glucocorticoids do not diffuse in the systemic circulation due to the ocular barriers, whilst extraocular administration might influence endogenous corticoids metabolism through negative feedback on the HPA^16^. Several studies have measured systemic endogenous cortisol levels in patients with CSCR with variable and contradictory results, but hair cortisol, that best represents systemic cortisol exposure several months prior analysis, was not elevated in a large cohort of patients with chronic CSCR^17^, questioning the generally admitted hypothesis that patients with CSCR might have chronically higher systemic cortisol levels. In line of this observation, in a cohort of patients with Cushing syndrome, we did not find increased occurrence of CSCR^18^, but whether ocular corticoids are elevated in eye with CSCR and whether the systemic levels of ocular corticoids reflect the systemic levels is unclear.

The adrenal gland produces gluco and mineralocorticoids hormones, grouped together under the term steroidome, the study of which helps to understand abnormalities in corticosteroid metabolism in a more complete and in-depth way than measuring cortisol alone, whose nychthemeral variations and dependence on stress make interpretation difficult^19^. The development of liquid chromatography coupled to mass spectrometry has emerged as the technique of choice for the quantitative analysis of the steroidome in biological matrices^20^.

In the eyeball, several studies have shown that cortisol levels in the aqueous humor contribute to the maintenance of immunological privilege. Different cortisol levels were measured in the aqueous humor depending on the analytical method, around 3 ng/ml (1.6–4.3 ng/ml) using Elisa^21^ and between 18 and 30 ng/ml using RIA^22, 23^. We previously showed that aldosterone was below detectable levels in human ocular media (using RIA, and mass spectrometry) and that level of cortisol was not significantly different in the aqueous humor and vitreous from different individuals, around 3–6 ng/ml ^24, 25^. Interestingly Knisely et al.^23^ reported undetectable levels of the cortisol-binding globulin (CBG) in the aqueous humor, postulating that cortisol is only in its free form in the human aqueous humor, contrarily to the serum in which 90–95% of cortisol is bound to CBG or albumin. Some other studies indicated that cortisol could be produced by corneal epithelial cells^26^ and by the retinal pigment epithelium^27^ suggesting that a local metabolism of corticoid takes place within the eye.

Corticoids are transcription factors that bind to both the gluco (GR) and the mineralocorticoids receptors (MR), both expressed in the human RPE/choroid and in the retina^28^. Based on animal and cell studies, we previously hypothesized that imbalance in the GR/MR pathways, towards MR pathway overactivation could be involved in retinal disease pathogenesis^25, 29, 30^. We then proposed to use oral MR antagonists to treat chronic CSCR^30–32^ and restore the receptors activity imbalance, which led to variable results^33, 34^. But, whether ocular corticoids are deregulated in CSCR is unknown and there is yet no evidence that MR is overactivated in eyes with CSCR.

More recently, in a rat model, we showed that imbalance towards MR pathway activation in the RPE/choroid occurred after chronic systemic dexamethasone exposure, which induced hypothalamo-pituitary axis negative feedback and subsequent reduced systemic and ocular levels of cortisol, showing simultaneous regulation of the corticoid receptors together with their ligands^35^.

In humans, the ocular steroidome and its relations with the systemic steroidome and its nychthemeral variations has not been fully explored and, the metabolism of ocular steroids has not been analyzed in the eyes with CSCR.

In this context, we have used a liquid chromatography coupled to tandem mass spectrometry method, already validated in aqueous humor and vitreous matrices^24, 25, 28^ to analyze the steroidome in the aqueous humor of patients undergoing cataract surgery and its relation with age, sex and time of the day. We then analyze simultaneously the aqueous humor and serum steroidome of healthy patients undergoing cataract surgery to study the link between ocular and systemic steroidome. Having observed that systemic steroidome was not reflecting the ocular steroidome, we analyzed the aqueous humor steroidome of a group of patients with complex and long lasting CSCR to explore potential local dysmetabolism. Finally, based on the observation that in the aqueous of eyes with complex and chronic CSCR, there could be a deficiency in activation of the glucocorticoid pathway, we present two cases of CSCR irresponsive to other treatments, who responded to topical glucocorticoids.

## Patients and methods

### Ethic statement

This research was conducted in compliance with the tenets of the Declaration of Helsinki and was approved by French by local Ethics Committee or “Comité de Protection des Personnes” (CPP). The collection and storage of human aqueous humors and vitreous collected during surgery was approved by committee CPP Ile de France 1 (N°2016-nov-14390). The collection of both serum and aqueous humor at the time of surgery is part of the Bioneoret study, which was granted approval by the by local Ethics Committee or “Comité de Protection des Personnes CPP IDF8 on the 16/01/2020 and on the 11/01/2022, authorized by the French authorities (ANSM), and registered in a public trials registry (EudraCT 2018–102,099-46, Clinical Trail NCT04439708, promoter Inserm). All patients have signed an informed consent form to be included in the study.

### Patients and samples

#### Control group

Fifty aqueous humors from 50 individuals operated for cataract surgery were analyzed to evaluate correlation of corticoids ocular levels with age, sex and time of surgery. Amongst them, twenty-seven patients had simultaneous serum and aqueous humor sampling. All cataract surgery patients only had topical anesthesia and dilation of the pupil was induced after aqueous humor retrieval by the intracameral injection of Mydrane® (Thea, Clermont-Ferrand, France). Surgery was performed between 8am and 6 pm. To evaluate whether levels of corticoids in the aqueous humor correlates with levels in the vitreous, nine other patients who underwent combined cataract and membrane peeling surgery were included. Their aqueous and vitreous humor were sampled at the same time. None of the patients had diabetic retinopathy, uveitis or age-related macular degeneration or myopia > 6D or had been treated with systemic or ocular glucocorticoids in the last 3 months.

#### CSCR Group

Fourteen patients with long-lasting complex central serous chorioretinopathy underwent cataract surgery in one eye, except one patient who underwent cataract surgery in both eyes at 2 months interval (15 AH were analyzed). All patients had pachychoroid features (pachyvessels, attenuation of the choriocapillaris), epitheliopathy and subfoveal choroidal thickness measured on enhanced depth imaging spectral domain optical coherence tomography superior to 350 µm. None of the patients were treated with any GC and none of them had active CSCR with SRF at the time of surgery.

#### Samples

Undiluted aqueous humor (at least 70 µl) was sampled at the beginning of cataract surgery before pupil dilation, vitreous biopsy was sampled before infusion opening to avoid any dilution and the ocular media were immediately frozen at − 80 °C until analysis. The serum was immediately prepared and then stored at − 80 °C until analysis.

### Corticosteroid profiling in human ocular and serum samples

#### Steroid hormone analysis

Steroid concentrations in plasma samples were measured by liquid chromatography coupled to tandem mass spectrometry, as previously reported^24^. Briefly, from a 200 µl plasma sample spiked with a mix of deuterated internal standards, steroids were purified by a protein precipitation procedure using zinc sulphate and methanol solutions followed by a solid phase extraction step (OASIS, Waters, Guyancourt, France). Aldosterone (ALDO), corticosterone (B), 11-dehydrocorticosterone (A), 11-deoxycorticosterone (DOC), progesterone (P4), 17-hydroxyprogesterone (17-OHP), 11-deoxycortisol (S), cortisol (F), cortisone (E), dehydroepiandrosterone sulphate (DHEAS), androstenedione (Δ4A) testosterone (T) and dehydroepiandrosterone (DHEA) were resolved using a BEH C18 column coupled to a methanol-ammonium format gradient during fourteen minutes. Detection was performed on a Xevo TQS tandem mass spectrometer (Waters, Paris, France) equipped with an electrospray operating in positive mode except for DHEAS determination. Quantification was performed in the multiple reaction monitoring mode. For eyes media analysis, steroids were extracted from 50 to 100 µl of aqueous or vitreous humor as described above. The calibration curve samples, zero samples (only internal standards added) and blank samples (no standards added) were prepared in charcoal-treated plasma or in physiological solution for plasma or eyes media samples, respectively. The data were fit to a linear least square regression curve with a weighing index of 1/*x*.

Calibration ranges, limit of quantification (LOQ) and between-run coefficients of variation (%) for individual steroids in both media are shown in Table 1.

**Table 1:** Calibration ranges, limit of quantification (LOQ) and between-run coefficients of variation (%) for individual steroids in serum and aqueous humor.

Analyte	Plasma matrix			Aqueous humor matrix		
	Calibration range (ng/mL)	LOQ (ng/mL)	Between-day CV (%)	Calibration range (ng/mL)	LOQ (ng/mL)	Between-day CV (%)
Aldo	0.022–2.770	0.015	6.31	0.020–0.500	0.010	9.24
B	0.529–45.400	0.040	4.24	0.010–2.500	0.015	9.52
A	0.100–2.0	0.040	9.31	0.010–1.000	0.015	10.81
DOC	0.044–3.150	0.020	11.05	0.020–1.000	0.020	13.24
P4	0.156–24.300	0.060	6.00	0.100–1.000	0.020	9.52
F	10.1–278.0	0.060	3.92	0.500–10.000	0.040	7.81
E	1.01–20.50	0.050	8.07	0.250–10.000	0.040	9.05
S	0.105–14.10	0.040	6.48	0.020–2.500	0.025	8.06
17-OHP	0.094–14.200	0.040	8.12	0.025–2.500	0.020	9.31
DHEAS	110–5971	50	9.46	10–2500	25	8.07
Δ4A	0.185–13.500	0.040	8.41	0.050–2.000	0.025	9.48
T	0.056–11.7	0.025	5.89	0.020–1.500	0.010	7.14
DHEA	0.875–57.8	0.150	8.52	0.050–5.000	0.075	10.46

*B* corticosterone, A 11-dehydrocorticosterone, *DOC* 11-desoxycorticosterone, *P4* progesterone, *F* cortisol, *E* cortisone, *S* 11-desoxycortisol, *17-OHP* 17-OH progesterone, *DHEAS* dehydroepiandosterone sulfate, *Δ4A* delta4-androstenedione, *T* testosterone, *DHEA* dehydroepiandosterone.

**Figure 1:**
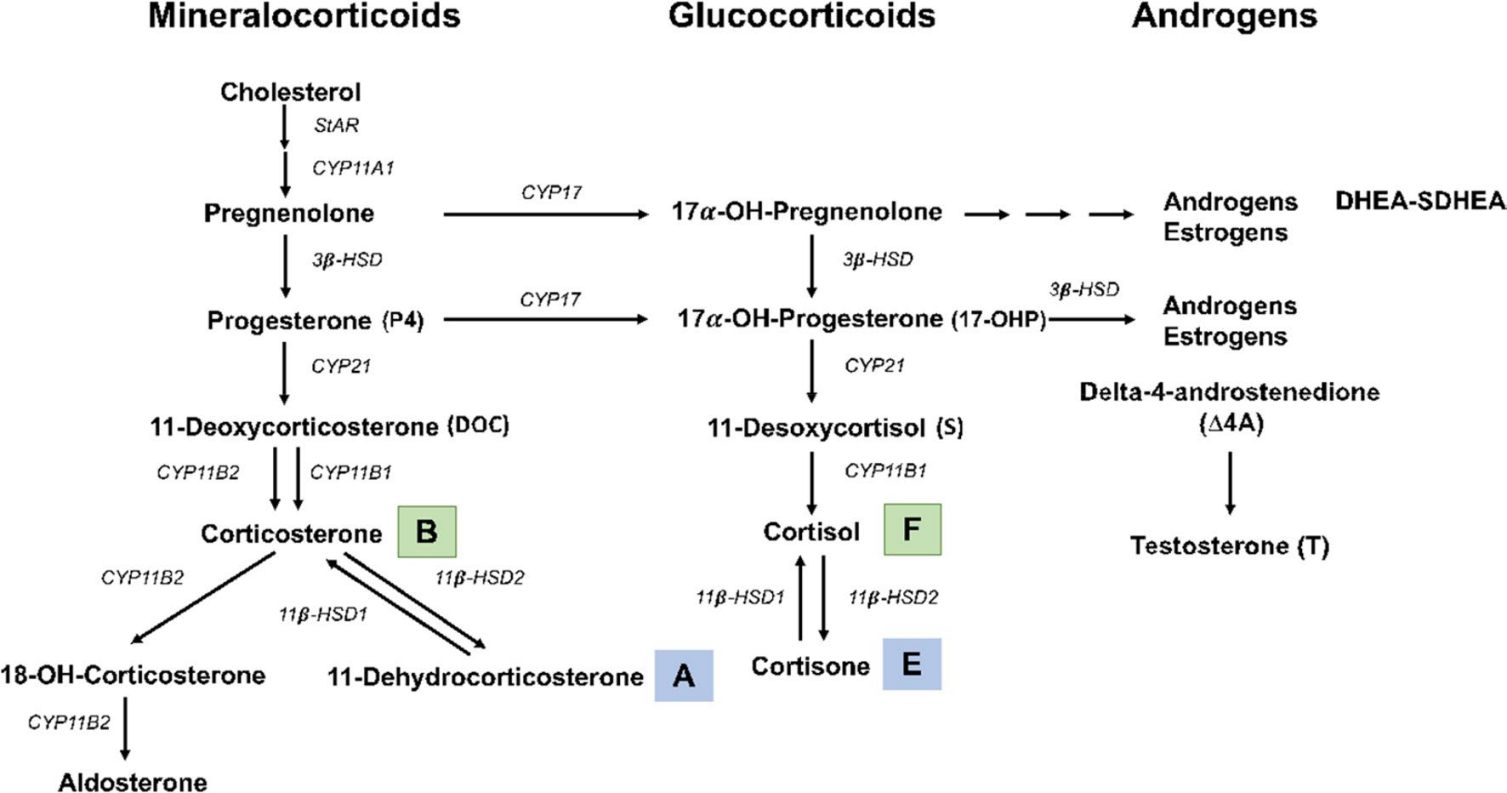
Corticoid metabolism. Corticoid metabolic pathways in the adrenal gland leading to glucocorticoids, mineralocorticoids and androgens synthesis. In green, F (cortisol) and B (corticosterone) are active forms, they are converted by the 11β-HSD2 to cortisone (E) and 11-dehydrocorticosterone (A), in blue, that are inactive forms with low affinity for the glucocorticoid receptor.

The 11-β-hydroxysteroid dehydrogenase isozyme 2 (11β-HSD2) is an MR protecting enzyme that oxidizes the active cortisol to the inactive metabolite cortisone, thus preventing illicit MR activation. By determining the product-to-substrate ratio (cortisone/cortisol, E/F), the 11β-HSD2 activity can be evaluated. Figure 1 shows the metabolic pathway of corticoid production in the adrenal gland with their nomenclature.

### Statistics

Quantitative data was tested for normality with the Shapiro–Wilks tests. They are reported as means with standard deviations (SD) when normally distributed and medians with interquartile ranges (IQR) when non-normally distributed. Data were compared between the two groups using Student t-test for normal distributions and Mann–Whitney U-tests for non-normal distributions. Correlation between two quantitative variables was evaluated using Spearman test. Statistical analyzes were performed using GraphPad Prism (version 5.0f., GraphPad Software). *p*-values < 0.05 were considered statistically significant.

This study has been conducted following another pilot study in which we have measured the levels of cortisol in ocular media of control and diabetic eyes, showing significant difference in diabetic patients^24^, allowing to calculate the number of patients needed to find a difference between a control group and a diseased group using the biostatgv.sentiweb.fr software.

## Results

### Patient characteristics

#### Control group

Fifty aqueous humors from 50 individuals operated for cataract surgery were analyzed to evaluate correlation of corticoids ocular levels with age, sex and time of surgery. There were 25 men and 25 women. The mean age was 73 ± 14.7 years [29–104 years]. Among this cohort, 27 patients (14 women and 13 men had simultaneous serum and aqueous humor sampling. Their mean age was 72 ± 13 years [29–92]. To evaluate whether levels of corticoids in the aqueous humor correlate with levels in the vitreous, nine patients (5 men and 4 women) who underwent combined cataract and membrane peeling surgery were included. Their mean age was 62.2 ± 12.7 years [45–82].

#### CSCR group

Fourteen patients (12 men and 2 woman) with long-lasting central serous chorioretinopathy (CSCR) (> 10 years, mean 23 ± 14 years) underwent cataract surgery in one eye, and one patient underwent cataract surgery in both eyes at 2 months interval. The CSCR patients were long-lasting complex cases with alteration of the retinal pigment epithelium associated with signs of pachychoroid and recurrent episodes of subretinal fluid during the follow-up period. Seven CSCR patients had a history of GC intake. Three patients had been treated with GCs for asthma. One patient received systemic GC in a context of allergy. Two patients received intra articular injection of GC (1 patients for discopathy and 1 patient for shoulder arthritis). One patient had an history of nasal inhalation of GC. Three patients had a history of argon-laser photocoagulation on leaky spots several years ago and had been patients been treated by verteporfin photodynamic therapy at least 1 year before the surgery. Another patient had been treated with verteporfin photodynamic therapy several years before the cataract surgery. Type 1 quiescent choroidal neovascularization was suspected in two eyes based on flat irregular pigment epithelium detachments associated with flow on optical coherence tomography angiography but without late phase hyper fluorescence on indocyanine green angiography. No sign of CNV activity was present. The mean subfoveal thickness was 475 ± 100 µm [377–693 µm]. None of the patient had subretinal fluid at the time of cataract surgery. These patients can thus be classified as complex CSCR^36^, not active at the time of AH sampling.

### Serum steroidome

**Figure 2:**
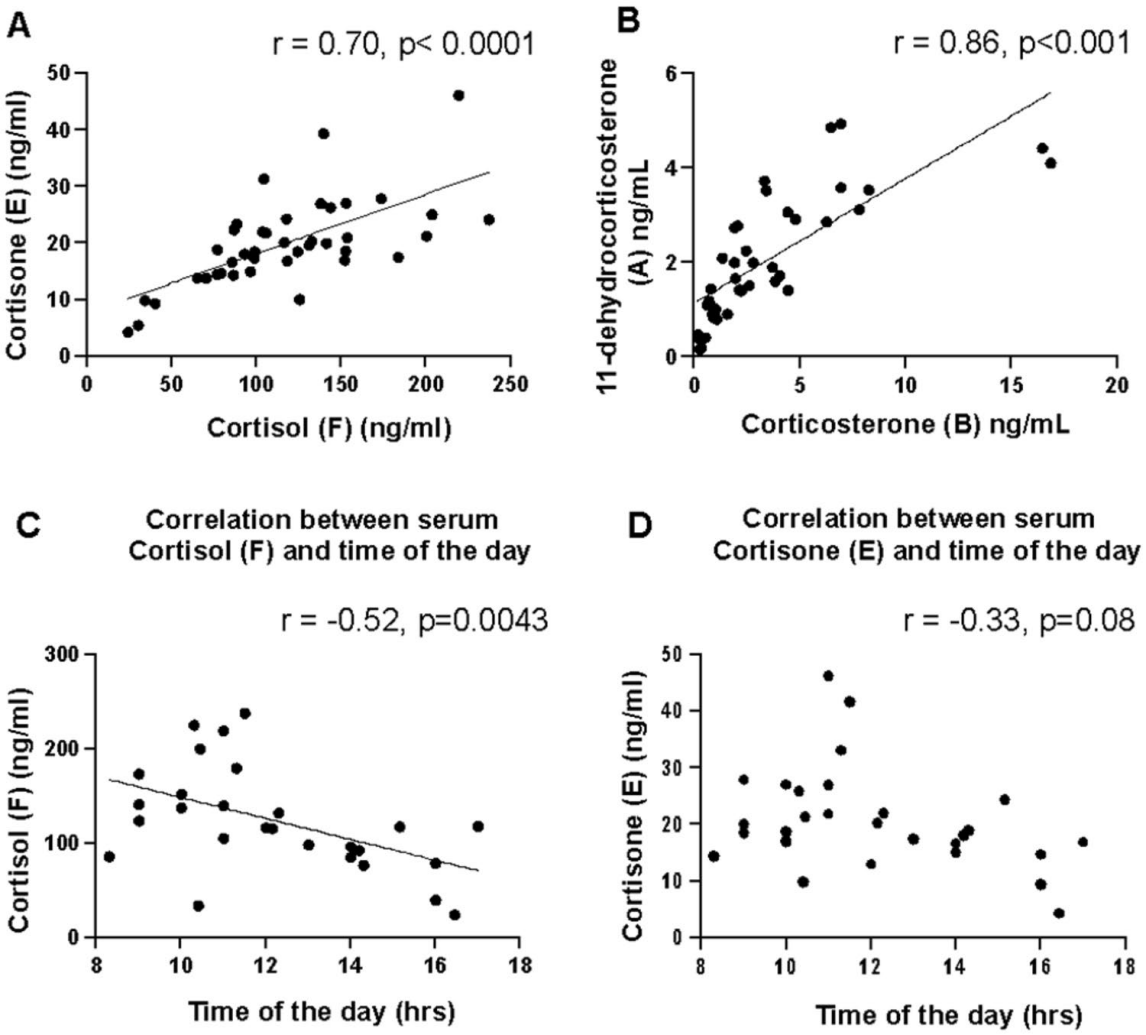
Corticoids in the serum. (**A**) Correlation between cortisol (F) and cortisone (E) levels (ng/ml) in the serum. (**B**) Correlation between corticosterone (B) and 11-dehydrocorticosterone (A) levels (ng/ml) in the serum. (**C**) Correlation between cortisol (F) level (ng/ml) and the time of the day (hours). (**D**) Correlation between cortisone (E) level (ng/ml) and the time of the day (hours).

All the analyzed corticoids were measured in the serum (Table 2) and the results are summarized in Fig. 2.

**Table 2:** Steroidome in the serum expressed in ng/ml.

Corticoids in serum	F	E	A	B	17-OHP	Δ4A	E/F	A/B
Mean ± SD	133.0 ± 48.6	21.3 ± 7.5	2.33 ± 1.69	4.41 ± 4.93	0.31 ± 0.22	0.62 ± 0.29	0.25 ± 0.05	1.33 ± 0.57
Min- Max	34.1–237.0	9.9–46.2	0.19–6.94	0.35–16.8	0.064–0.693	0.31–1.33	0.11–0.29	0.67–2.39

Corticoids in serum	S	DOC	P4	Aldo	T	DHEA	SDHEA	
Mean ± SD	0.22 ± 0.18	0.131 ± 0.293	0.116 ± 0.250	0.075 ± 0.065	2.62 ± 2.80	9.3 ± 5.6	1139 ± 597	
Min- Max	0.03–0.61	0.015–0.960	0.01–1.183	0.015–0.264	0.12–8.5	1.5–19.63	394.5–2176

As expected, the systemic steroidome shows variation of cortisol levels with the time of sampling and a positive correlation between the active and inactive forms of the gluco (F,E) and mineralocorticoids (B, A) which represents the activity of the 11β-hydroxysteroid dehydrogenase type 1 and 2 enzymes.

There was a positive statistically significant correlation between serum cortisone (E) and serum cortisol (F) levels (Spearman r = 0.70, *p* < 0.0001) and between serum 11-dehydrocorticosterone (A) and corticosterone (B) (Spearman r = 0.86, *p* < 0.0001) (Fig. 2A,B). There was a correlation between the time of sampling and cortisol level in the serum (r = − 0.52, *p* = 0.0043), with higher levels measured in the morning (Fig. 2C). Serum cortisone, the inactive form of cortisol, did not correlate significantly with the time of sampling (r = − 0.33, *p* = 0.08) (Fig. 2D). In the serum, mean aldosterone level was 0.075 ± 0.065 ng/ml [0.015–0.264] and it was not correlated with the time of the day (Spearman r = 0.38, *p* = 0.1). The systemic steroidome is shown in Table 2.

### Aqueous humor steroidome

In the aqueous humor, aldosterone, progesterone (P4), 11-deoxycorticosterone (DOC), 11-deoxycortisol (S), testosterone (T), DHEA dehydoepiandrosterone (DHEA) and Sulfate of dehydroepiandrosterone (SDHEA) were below limit of quantification in all samples.

The composition of the ocular steroidome is therefore limited compared to that of serum, demonstrating selective mechanisms in the passage of corticoids from the circulation to the aqueous humor as shown at the blood–brain barrier^37, 38^.

Table 3 shows the ocular steroidome measured in the aqueous humor. The 17 hydroxyprogesterone (17-OHP) was measurable in AH with a mean level of 0.048 ± 0.016 ng/ml [0.035–0.083]. Delta4-androstenedione (∆4A) was above detectable level in 70% of the samples with a mean level of 0.031 ± 0.026 ng/ml.

**Table 3:** Steroidome in the aqueous humor expressed in ng/ml.

Corticoids in AH	F	E	A	B	17-OHP	D4	F/E	B/A	E/F	A/B
Mean ± SD	5.05 ± 1.34	0.987 ± 0.438	0.10 ± 0.07	0.18 ± 0.09	0.048 ± 0.016	0.031 ± 0.026	6.087 ± 3.2	0.9 ± 1.5	0.195 ± 0.078	0.69 ± 0.52
Min- Max	1.15–7.80	0.01–1.71	0.008–0.294	0.05–0.38	0.035–0.083	0.001–0.098	2.4–17	0.014- 7	0.06–0.37	0.03–2.5

**Figure 3:**
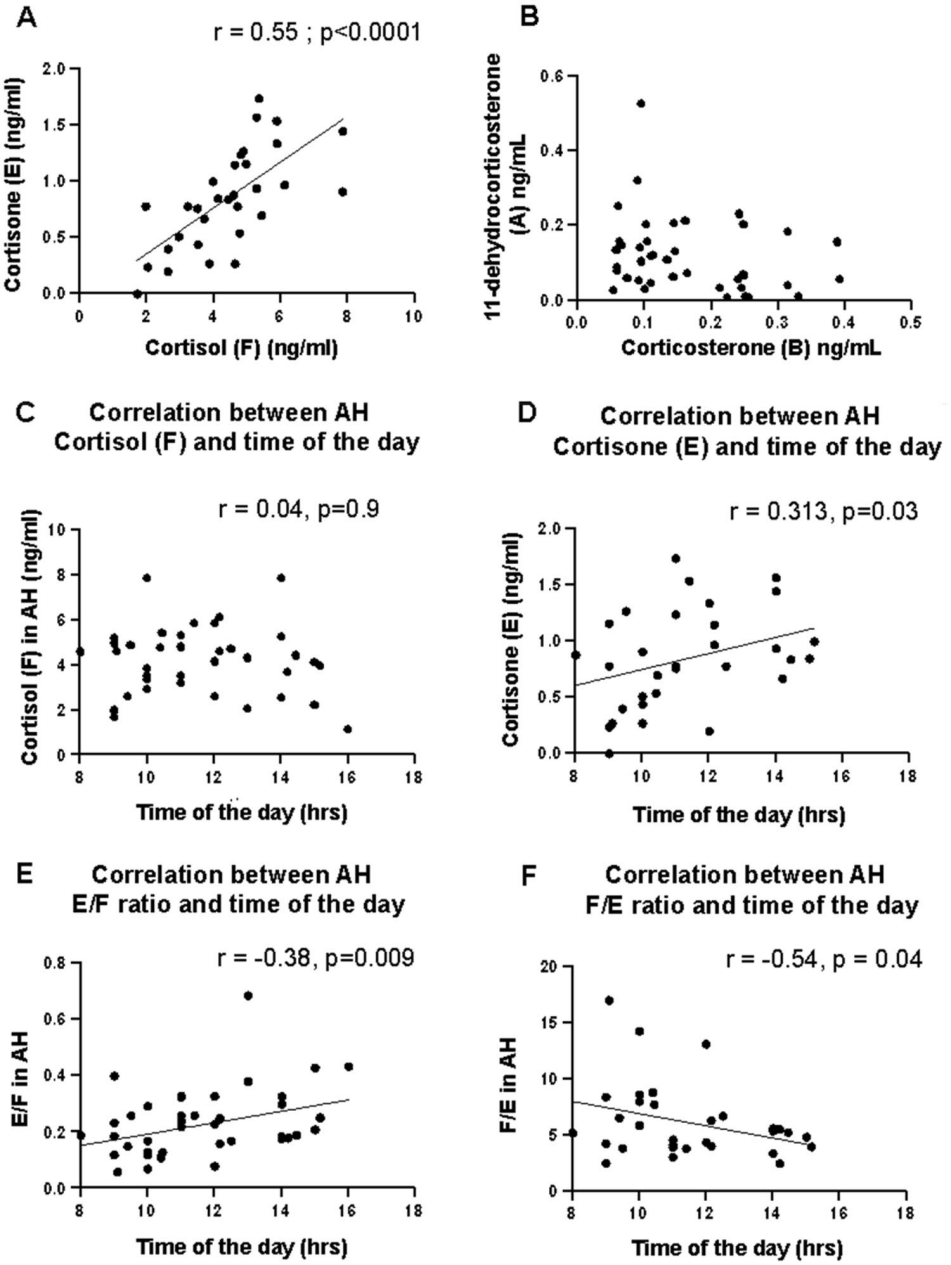
Metabolism of corticoids in the aqueous humor (AH). (**A**) Correlation between cortisol (F) and cortisone (E) levels (ng/ml), (**A**) correlation between corticosterone (**B**) and 11-dehydrocorticosterone (**A**) levels (ng/ml), (**C**) correlation between the time of the day and the level of cortisol (F) in the AH, (**D**) significant and positive correlation between cortisone (E) level (ng/ml) and the time of the day (r = 0.3, *p* = 0.03), (**E**) significant influence of the time of the day on the E/F in the AH, (**F**) significant influence of the time of the day on the F/E ratio in the AH.

In the aqueous humor, the mean E level was 0.987 ± 0.438 ng/ml [0.01–1.71] and the mean F level was 5.05 ± 1.34 ng/ml [1.15–7.80] (n = 50) and intraocular cortisol (F) and cortisone (E) levels correlated positively (Spearman r = 0.55, *p* < 0.0001). The F/E ratio which reflects the activity of the 11β-HSD enzymes demonstrates a prevalence of the active cortisol form that activates the GR pathway. The mean corticosterone (B) level was 0.18 ± 0.09 ng/ml [0.05–0.38] and the 11-dehydrocorticosterone (A) level was 0.10 ± 0.07 ng/ml [0.008–0.294] but there was no correlation between A and B levels in the AH (Spearman r = 0.1, *p* = 0.4) (Fig. 3A,B) indicating that MR pathway can be activated by corticosterone in the eye like in the brain^39^.

### Influence of the circadian rhythm on ocular corticoid levels

Reflecting the circadian clock, systemic cortisol levels fluctuate between a peak in the early hours of the morning to a minimal at midnight, as observed in the declined serum levels of individuals sampled in the evening as compared to the morning. In the aqueous humor, there was no significant correlation between the time of the day and the level of cortisol (F) [Fig. 3C] (r = 0.04, *p* = 0.9), but there was a positive correlation between time of the day and the cortisone levels (Spearman r = 0.313, *p* = 0.03) [Fig. 3D], with surprisingly higher cortisone levels in the evening as compared to the morning. Consequently, E/F ratio significantly increased with the time of the day (Spearman r = 0.38, *p* = 0.009) and F/E decreased with the time of the day (r = − 0.54, *p* = 0.04) (Fig. 3E,F) showing that the ratio of active cortisol is lower in the evening not because of decreased cortisol levels like in the serum but due to higher either activity of the 11β-HSD2 enzyme that converts cortisol into inactive cortisone or to higher cortisone entry at later times in the day. There was no influence of the time of sampling and the aqueous levels of A, B and B/A (*p* > 0.005) (not shown).

### Influence of age on corticoids ocular levels

There was no correlation between the age of the patients at the time of surgery and the level of cortisol (F) (Spearman r = 0.03, *p* = 0.833) or cortisone (E) in the aqueous humor (Spearman r = − 0.006, *p* = 0.966). There was no correlation between the age of the patients and the E/F ratio in the aqueous humor (Spearman r = − 0.07, *p* = 0.64) [not shown]. No correlation was found between A, B and A/B and the age of the patient as well (*p* > 0.05).

### Influence of sex on corticoids ocular levels

**Figure 4:**
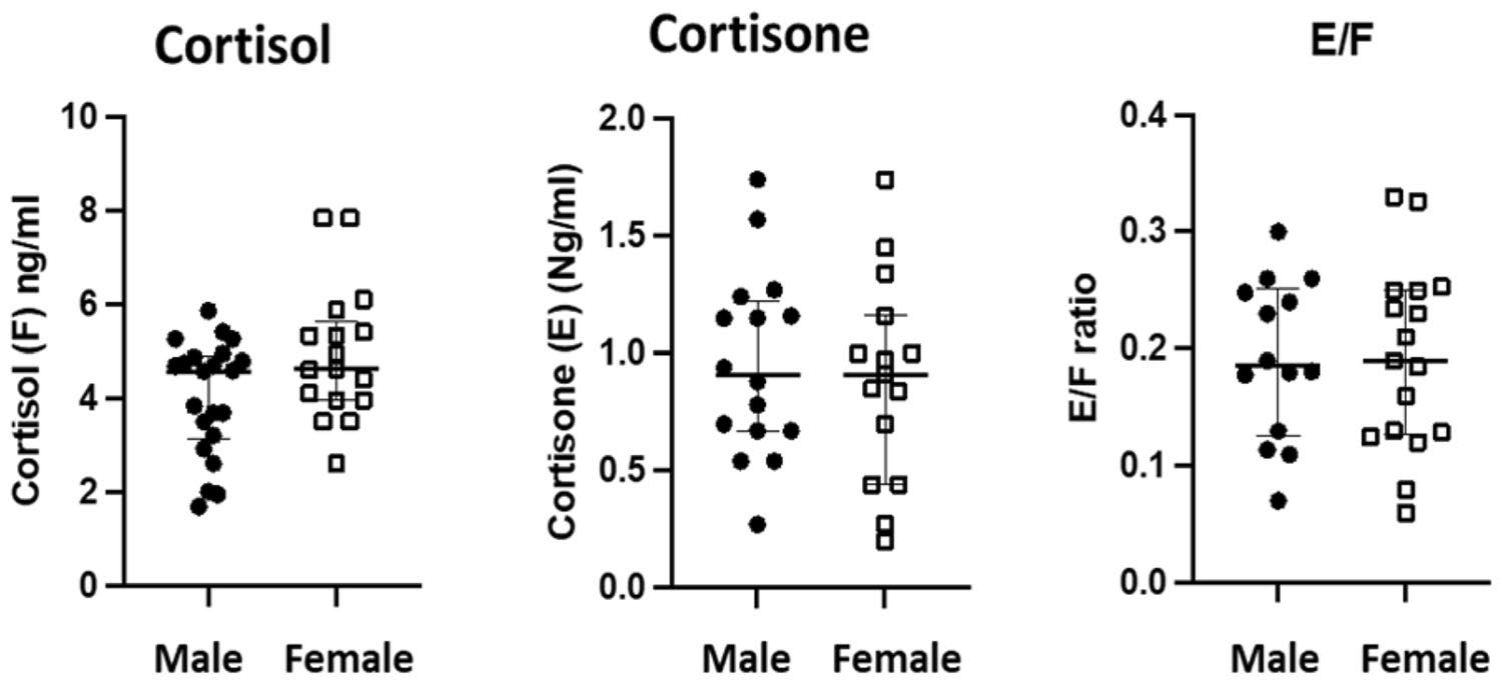
Influence of sex on the metabolism of glucocorticoids in the AH. No significant difference between male and female in the levels of cortisol (ng/ml) (**A**), cortisone (ng/ml) (**B**) and E/F ratio (**C).**

There was no significant difference between cortisol levels measured in the aqueous humor of women as compared to men (*p* = 0.39) (Fig. 4A), no difference was measured between cortisone levels (*p* = 0.89) (Fig. 4B) and E/F ratio and sex (*p* = 0.9) (Fig. 4C). No significant difference was found in A, B and A/B levels in the aqueous humor of male and females (not shown). Thus, sex did not influence the levels of gluco and mineralocorticoid hormones in the aqueous humor.

The 17-OHP (17-hydroxyprogesterone) levels were not significantly different between men and women (*p* = 0.28) but ∆4A, levels were significantly lower in women as compared to men (0.01 ± 0.04 vs. .019 ± 0.015 ng/ml, *p* = 0.0075) since it is an adrenal androgen. Pogesterone and testosterone were below detectable levels in all samples and, ∆4A was the only androgen hormone measured in the aqueous humor.

### Correlation between corticoids levels in aqueous humor and vitreous (V)

**Figure 5:**
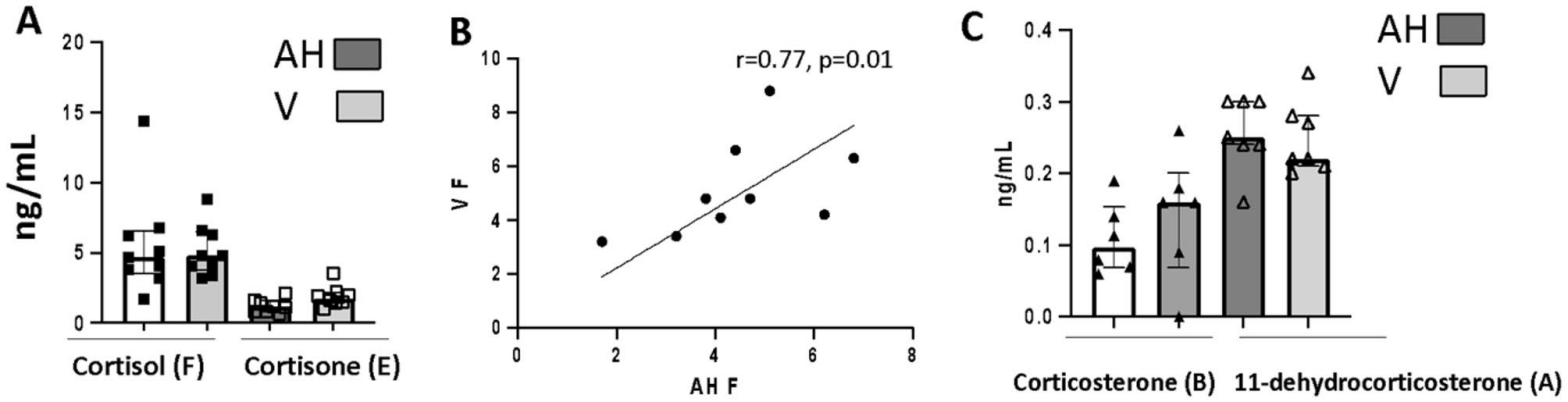
Comparison between gluco and mineralocorticoids in the AH and vitreous (V) of the same individual.

In a specific group of patients, we collected AH and undiluted V during the same surgery to evaluate whether levels of corticoids could differ between the two compartments. There was no significant difference between the level of cortisol (F) in the AH or in the V (4.4 vs. 4.8 ng/ml, *p* = 0.5) and the levels of F in the AH and V of a same patient were correlated (Spearman r = 0.77, *p* = 0.01) (Fig. 5A,B). There was also no significant difference between the level of cortisone (E) in the AH and in the V (Fig. 5A) (1.2 vs. 1.9 ng/ml), but there was no significant correlation between E level in the AH and V of the same patient (*p* = 0.25). The levels of corticosterone (B) and of 11-dehydrocorticosterone (A) were not significantly different in the AH and V of the same patient (Fig. 5C). A/B and E/F ratio were not significantly different in the AH and V. Levels of corticoids did not differ significantly in the AV and V from the same patient suggesting that sampling the AH is representative of V levels, at least in eyes without significant ocular pathology.

### Correlation between serum and ocular corticoids levels

**Figure 6:**
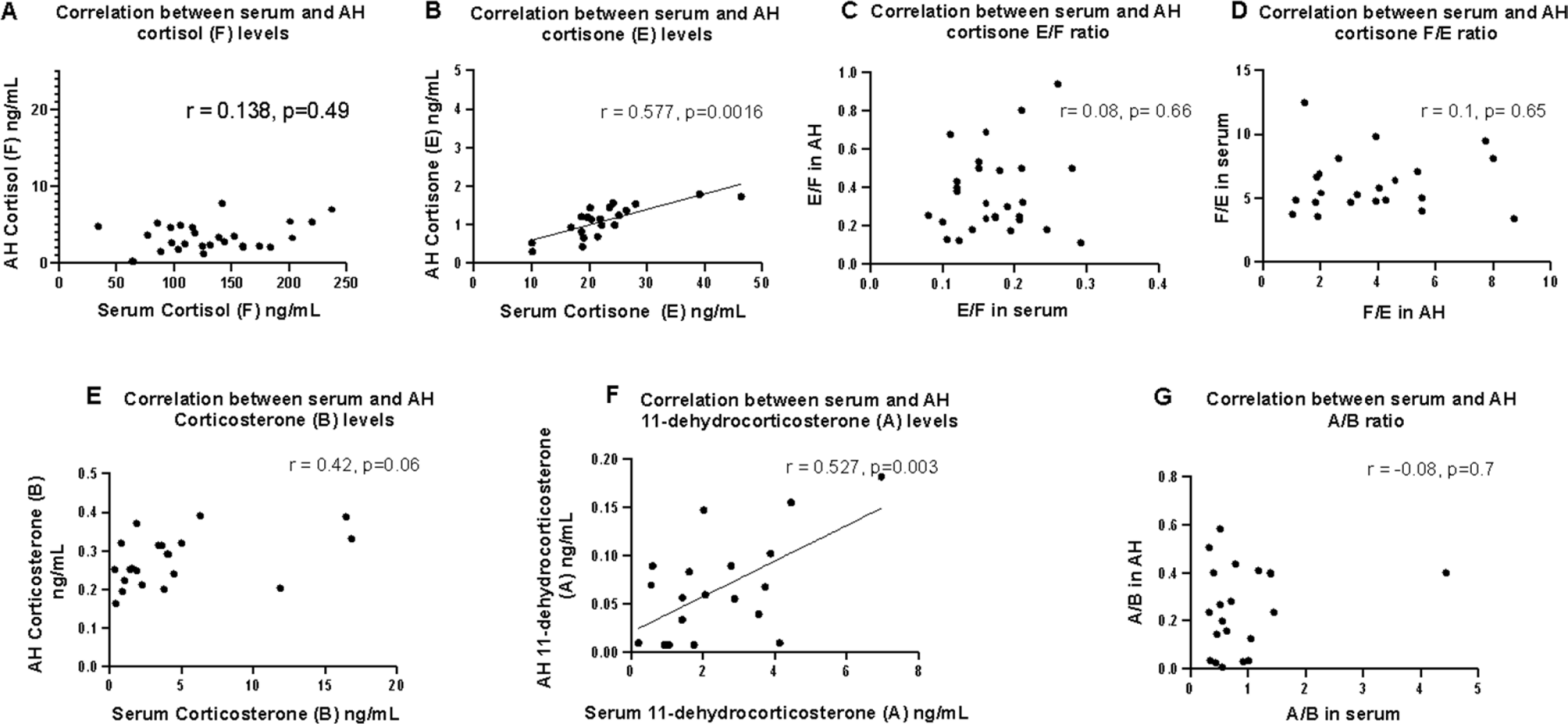
Comparison of corticoids metabolism in the AH and in the serum. (**A**) No correlation between cortisol (F) level (ng/ml) in the serum and in the AH (r = 0138, *p* = 0.49), (**B**) significant and positive correlation between cortisone (E) level (ng/ml) in the serum and in the AH (r = 0.57, *p* = 0.0016). (**C**) No correlation between the E/F ratio in the serum and in the AH. (**D**) No correlation between the F/E ratio in the serum and in the AH. (**E**) No correlation between corticosterone (B) level (ng/ml) in the serum and in the AH (r = 0.42 *p* = 0.06) (**F**). **S**ignificant and positive correlation between 11-dehydrocorticosterone (A) level (ng/ml) in the serum and in the AH (r = 0.52, *p* = 0.003). **G.** No correlation between the A/B ratio in the serum and in the AH.

To evaluate the link between serum and aqueous humor steroidome, we sampled simultaneously the aqueous humor and the blood of patients undergoing uncomplicated cataract surgery. There was no correlation between serum cortisol and aqueous humor cortisol levels (Spearman r = 0.138, *p* = 0.49) (Fig. 6A). No more than 10% of serum cortisol was measured in the aqueous humor, indicating that active mechanisms control the entry of cortisol from the serum into the eye, preventing the cortisol level from following the increase in circulating cortisol. Thus, spikes in circulating cortisol do not result in an increase in ocular cortisol.

On the other hand, there was a positive and significant correlation between serum and aqueous humor cortisone (E) levels (Fig. 6B) (Spearman r = 0.577, *p* = 0.0016), although levels of cortisone in the aqueous humor also remained low, around 5% of serum cortisone levels. This could indicate that the inactive form of cortisol (F), cortisone (E), could be transferred more easily from the serum into the aqueous humor, although being also a ligand of efflux transporters. The E/F ratio witnesses the activity of 11β-HSD enzymes and the metabolism of cortisol. There was no correlation between corticoid metabolism in the serum and in the AH (Fig. 6C,D).

Levels of corticosterone (B) in the AH and in the serum did not correlate (Spearman r = 0.42, *p* = 0.06), but the levels of dehydrocorticosterone (A), the inactive form correlated positively in the AH and serum (r = 0.527, *p* = 0.003) although less than 10% of serum A and B levels were measured in the AH, indicating that A and B entry into the eye is also regulated by efflux proteins (Fig. 6E,F). Like the E/F ratio, the A/B ratio in the serum and the AH did not correlate, indicating the activity of the 11-bHSD1 and HSD2 enzymes in the AH is independent from that in the serum (Fig. 6G).

### Corticoid levels in eyes with CSCR

**Figure 7:**
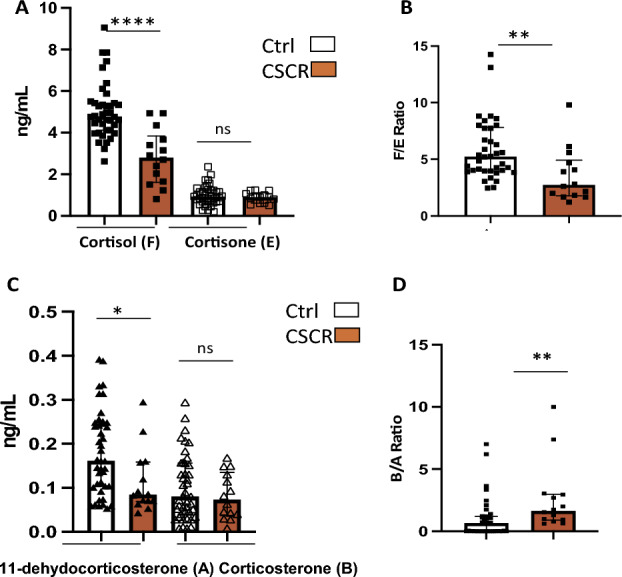
Comparison ocular steroidome in patients with CSCR and controls. (**A**) Levels in ng/ml of cortisol (F) (****p = 0.0001) and cortisone (E) (ns, p > 0.05) in control and CSCR eyes. (**B**) F/E ratio in control and CSCR eyes (**, *p* = 0.023). (**C**) Levels in ng/ml of corticosterone (B) (*p = 0.03) and 11-hydroxycorticosterone (A) in control and CSCR eyes. (**D**) B/A ratio in control and CSCR eyes (**, *p* = 0.0044).

As cortisol (F), corticosterone (B) and 11 deoxycorticosterone (A) levels in the aqueous humor correlate neither with the age or the sex of the individual, nor with the time of the day, we have compared F, B and A levels in the AH of 15 eyes with complex CSCR, not presenting subretinal fluid at the time of sampling, to those of the corticoids in the controls eyes (Fig. 7).

Since cortisone and the F/E ratio varied with time of the day, we compared first E and F/E with all control samples but also compared the 15 AH from CSCR eyes, that were all collected between 12 and 3PM with control samples, collected at the same time of the day (n = 25 time-matched controls). Cortisol level (F) was lower in CSCR eyes as compared to controls eyes (2.9 ± 1.2 vs. 5.0 ± 1.3 ng/ml, *p* = 0.0001) (Fig. 7A). Corticosterone (B) was significantly lower in CSCR than in controls eyes (0.11 ± 0.07 vs. 0.18 ± 0.09 ng/ml, *p* = 0.03). There was no significant difference in the E and A levels between CSCR and controls (0.99 ± 0.44 vs. 0.98 ± 0.46 ng/ml and 0.07 ± 0.06 vs. 0.10 ± 0.07 ng/ml, respectively, *p* = 0.5 for both). The B/A ratio was 3.36 ± 5.1 [0.6–10] in CSCR eyes and significantly higher than in control eyes (0.99 ± 1.56 [0.014–7], *p* = 0.018) (Fig. 7B). The F/E ratio was significantly lower in the AH of CSCR eyes (3.5 ± 2.3) as compared to all controls (6.1 ± 3.2) (*p* = 0.023) and it remained significantly lower as compared to time-matched controls (5.1 ± 2.3) (*p* = 0.01) (Fig. 7). We did not measure significant difference in the level of 17-OHP and no difference in the level of ∆4A when comparing only men CSCR (n = 12) with control males (n = 25).

Finally, since the mean age of CSCR patients was significantly lower than the one of controls and although we did not find any influence of age on the steroidome of control individuals, we then performed a sub analysis comparing the ocular steroidome of CSCR patients with the one of age-matched controls (*p* = 0.7, n = 15). In this subanalysis, cortisol (F) level was significantly lower in the CSCR group as compared to the age-matched controls (*p* = 0.0001), the cortisone (E) levels was not different (*p* = 0.8), the F/E ratio was significantly decreased (*p* = 0.04) showing similar results using this smaller group of controls (n = 15) and a trend towards a lower glucocorticoid pathway activation in eyes with CSCR.

#### Correlation between intraocular corticoids and subfoveal choroidal thickness in CSCR patients

In the group of patients with CSCR, subfoveal choroidal (SFCT) thickness was measured in the regular follow up protocol using EDI SD-OCT less than 8 days before the AH was collected. There was no correlation between subfoveal choroidal thickness and ocular F level (Spearman r = − 0.20, *p* = 0.46), E level (r = 0.11, *p* = 0.67), F/E (r = − 0.166, *p* = 0.55), A level (r = − 0.26, *p* = 0.37) and B level (r = 0.53, *p* = 0.06).

#### Case 1

**Figure 8:**
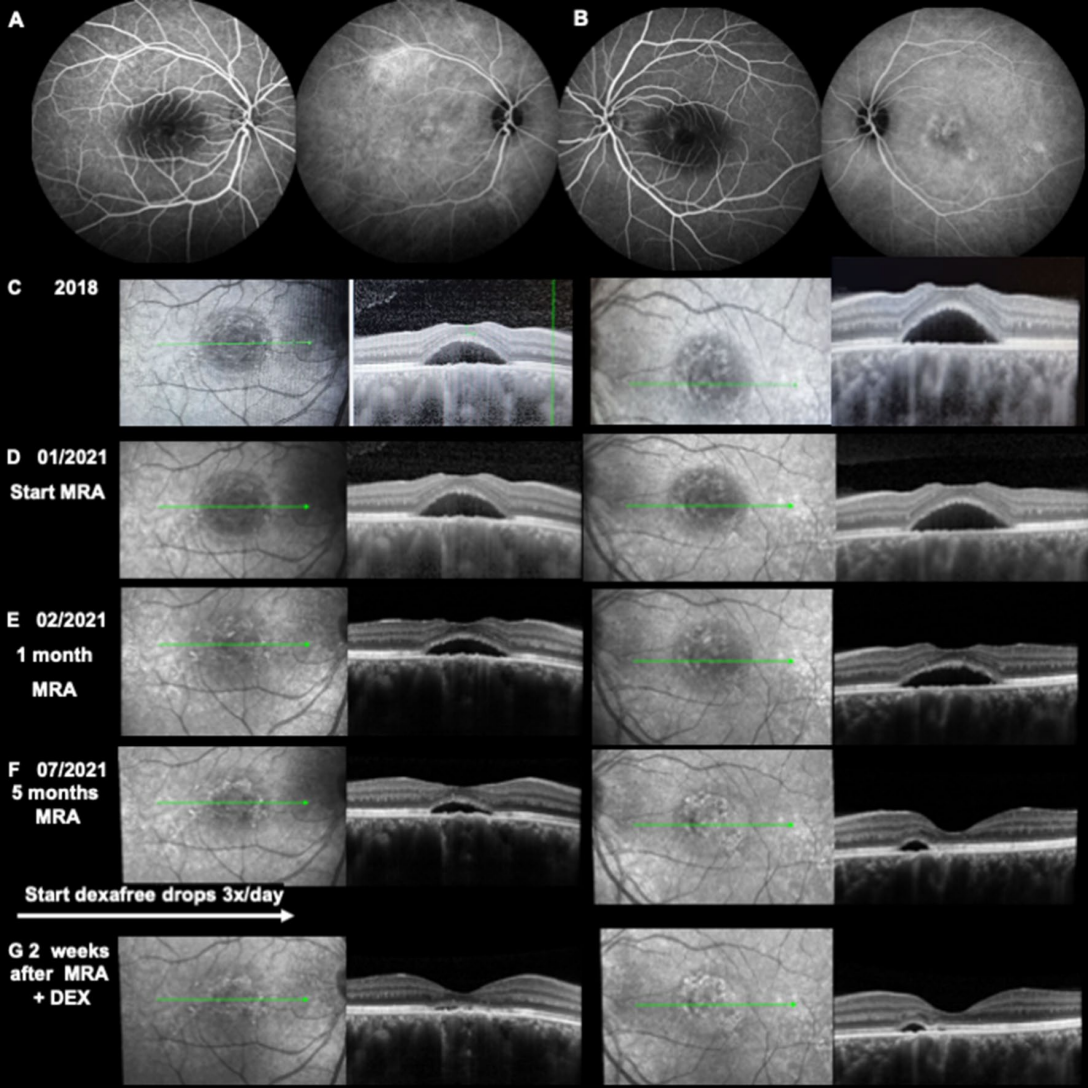
Multimodal imaging of a 32-year-old woman with bilateral chronic CSCR. (**A**) Intermediate phase at 4 min shows mild staining in the inferior foveal zone and moderate stippled hyperfluorescence at the level of the superior arcade on fluorescein angiography of the right eye, corresponding to a hyperfluorescent plaque on indocyanine green angiography (ICGA). The intermediate cliché at 7 min of the left eye, (**B**) shows a mild FA staining surrounding the foveal SRF pocket, corresponding to granular stippled hyperfluorescence on ICGA (**C–G**)**.** spectral-domain optical coherence tomography (SD-OCT) of the left and right eye during follow-up, respectively (**C**). The exam recorded in 2018 already showed a foveal neurosensory dome-shaped detachment with photoreceptor elongation and thickened choroid (**D–F**). Progressive SRF resolution after starting systemic mineralocorticoid antagonist therapy. The effect of the treatment on choroidal thickness cannot be evaluated without the enhanced depth imaging focus (EDI) (**G**). Additional topical dexamethasone treatment increased SRF reduction. Note the vascular enlargement with choriocapillary attenuation and signal hypertransmission underneath the residual neurosensory detachment and slight RPE elevation. No macular neovascularization was observed at that timepoint in both eyes (OCTA not shown).

Caucasian women, 32-year-old with a history of asthma since childhood, having being treated for years with inhaled and oral corticosteroids until she was 20 years old. She then periodically took inhaled corticoids. She did not present any other risk factors. In 2018, after psychological familial stress, she was diagnosed with bilateral CSCR and treated with half-fluence verteporfin photodynamic therapy (PDT) in both eyes in 2018 with transient efficacy as she reported recurrence less than 2 months after treatment. She was referred in November 2020 with bilateral serous macular detachment, similar to images she provided from 2018 (Fig. 8C).

**Figure 9:**
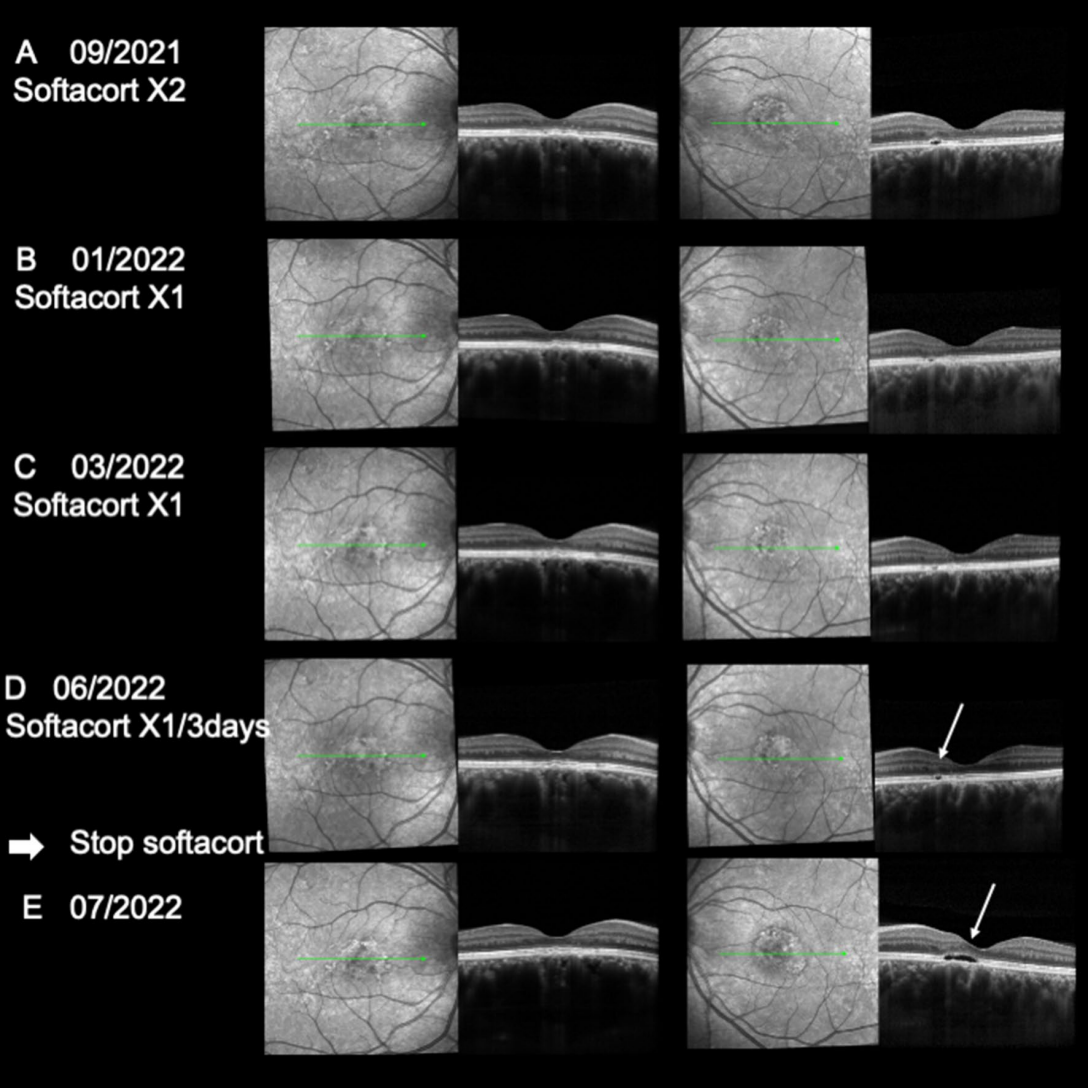
SD-OCT follow-up after glucocorticoid switch. (**A–C**) After hydrocortisone switch and slow tapering, a complete absence of CRF was documented, together with a good photoreceptor (PR) layer reconstitution and mild RPE irregularities. (**D**) Upon furtherly tapering hydrocortisone to 1 drop every 3 days, a minimal PR detachment was observed (white arrow) in the left eye. Glucocorticoid treatment cessation and close follow-up at 3 weeks **(E)** revealed an initial recurrence of the disease in the left eye corresponding to the same perifoveal area (white arrow).

At presentation, best corrected visual acuity was 8/10 on both eyes and the patient complained of micropsia and loss of contrast. On spectral domain optical coherence tomography (SD-OCT) B scan, subfoveal choroidal thickness was 680 µm in the RE and 516 µm in the LE with massive dilation of choroidal veins and effacement of the choriocapillaris. Macular subretinal fluid was present in both eyes (Fig. 8D). FA and ICG angiography performed in January 2021 showed a faint leaky site nasal to the fovea in the right eye and no leaky site on the left eye with mid-phase hyperfluorescent plaques more pronounced in the left than in the right eye along temporal vessels, at 7 min (Fig. 8A,B). No sign of choroidal neovascularization was found on ICG-A or on optical coherence tomography angiography (OCTA). Eplerenone 50 mg was introduced in January 2021 resulting in reduction of SRF at 1 and 3 months but without complete resolution of the SRF (Figs. 8E,F). Preservative free dexamethasone drops (twice per day) were introduced in July 2021 resulting in almost complete resolution of SRF at 15 days in both eyes (Fig. 8G). The patient was followed with complete resolution of SRF in both eyes and she was maintained on dexamethasone 1 drop/day without any raise in intraocular pressure for 2 months (Fig. 9A). The vision was 10/10 when she was switched to Softacort® 1 drop/day and remained free from recurrence until June 2022 (Fig. 9B,C), where an initial minimal increase in SRF appeared on OCT upon reduction of drop to 1 every 3 days (Fig. 9D). Recurrence of SRF was observed in the left eye 3 weeks after she had stopped all treatments (Fig. 9E).

#### Case 2

**Figure 10:**
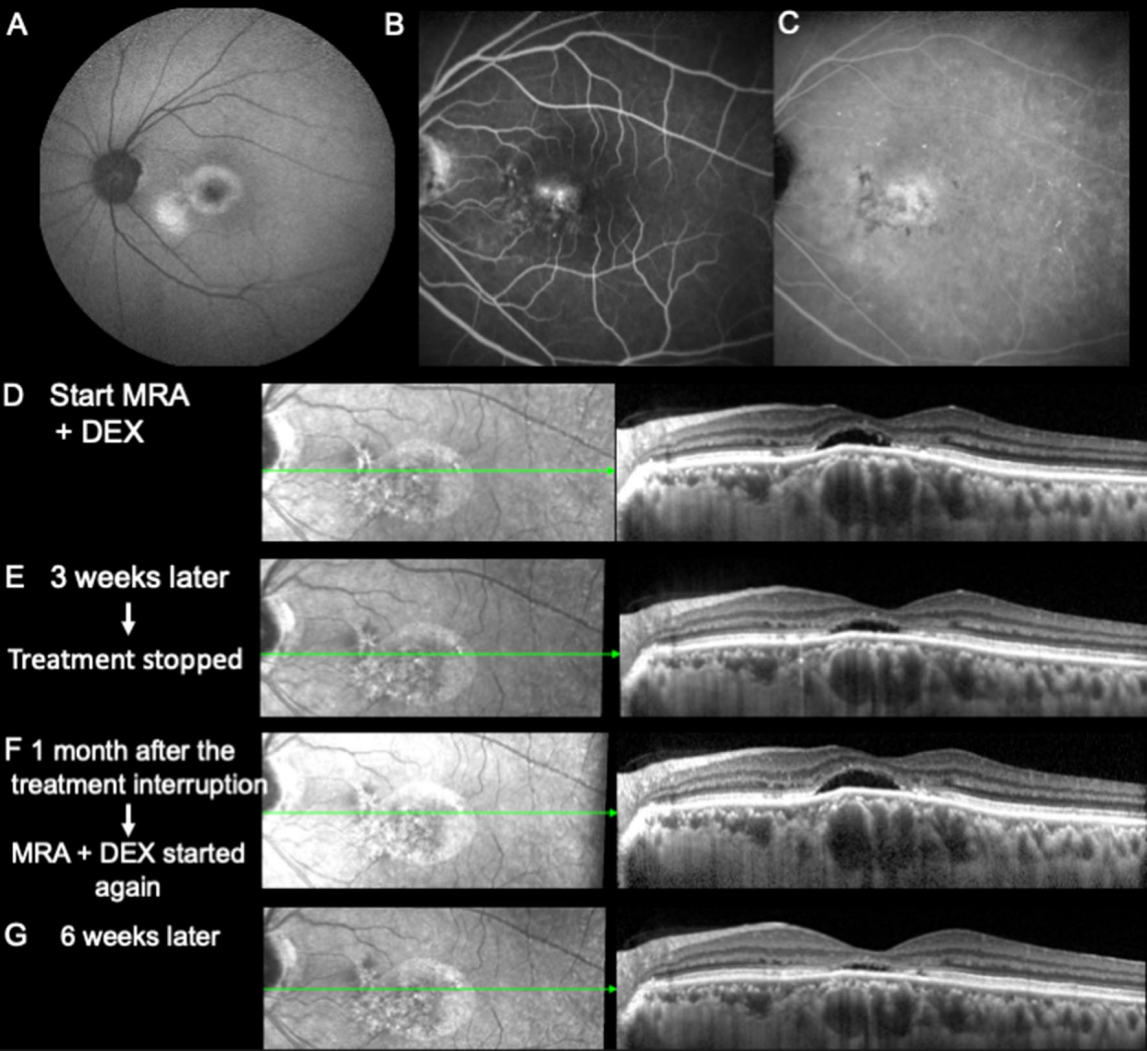
Multimodal imaging of the left eye (LE) of a 58-year-old male. (**A**) Fundus autofluorescence (FAF) showing perifoveal and inferior foveal hyperfluorescence. Intermediate FA (**B**) and ICGA (**C**) clichés at 5 min, showing stippled foveal hyperlfuorescence and granular irregular hyperfluorescence, respectively. (**D**–**G**) SD-OCT B-scans and infrared fundus imaging during the follow-up period.

58-years old Asian man, who is referred for unresolved and stable subfoveal fluid in the last 4 years, despite one PDT, 3 years ago. Visual acuity is 1/10. In the left eye, ICG-angiography showed no hyperfluorescent plaques or hyperpermeability signs and fluorescein angiography (FA) showed two juxta foveal and nasal leakage spots, not accessible to laser treatment (Fig. 10A–C). On SD-OCT, the dilated choroidal veins are overhung by a bulge of the pigmentary epithelium, but the choriocapillaris remains visible (Fig. 10D). Subfoveal choroidal thickness was 390 µm in the left eye and 350 in the right eye. In the left eye, foveal subretinal fluid was associated with thinning of the outer nuclear layer and shortening of the photoreceptor segments attesting of chronicity of the disease. At 3 weeks after a combination of eplerenone 50 mg and dexamethasone preservative free drop (3 times per day), there was a reduction of the subfoveal choroidal thickness from 390 to 350 µm. However, there was still SRF [Fig. 10E]. The patient discontinued all treatment by himself and came back 1 month later with increased SRF and increased subfoveal choroidal thickness to 380 µm (Fig. 10F). Six weeks after restoration of the initial treatment, SRF almost resolved and subfoveal choroidal thickness decreased to 290 µm, demonstrating the on–off effect of the treatment both on central macula thickness (CMT) and on choroidal thickness and veinous dilation (Fig. 10G). Vision remained low at 1/10.

## Discussion

In this study, we have used a highly sensitive and specific LC–MS/MS method, able to quantify steroids from the mineralocorticoid, glucocorticoids and androgen pathways ^20^ in less than 100 µl allowing to perform a complete profiling of corticoids in the ocular media of humans, called the steroidome. We performed several analyzes from control patients who underwent cataract surgery at different times of the day, a correlative analysis of serum and aqueous humor sampled at the same time and another correlative study to evaluate simultaneously the steroidome in AH and V from the same patients operated for combined cataract and membrane peeling surgery.

### Ocular steroidome reveals a local metabolism of corticoids

Whilst all tested corticoids were measured in the serum as expected, only 17-OHP, E, F, A and B were above limit of quantification in the aqueous humor in the gluco and mineralocorticoid pathways, confirming previous observation that aldosterone is not measurable in the eye, under normal conditions^24, 28^. Recently, one group analyzed corticoids using LC–MS/MS in aqueous humor and did measure aldosterone at level of 0.05 ng/ml^40^. We did not confirm this finding in our samples although limit of quantification for aldosterone is < 0.01 ng/ml with our method. In previous experiments, we also failed to measure aldosterone in ocular media from control individuals using other techniques, including radio-immunoassay. Although it cannot be excluded that depending on the sensitivity of the technique, very low levels of aldosterone could be detected, it can be ascertain that, like in the brain, the gluco and mineralocorticoid receptors, highly expressed in the ocular tissues^28, 41–43^ are mainly occupied by cortisol and by corticosterone that have similar to aldosterone, high affinity for MR^44^. In the choroid and at the basolateral side of the RPE, circulating aldosterone could eventually bind and activate MR, that is expressed in human endothelial cells of choriocapillaris, veins and arteries^45^ and in human RPE cells^25^, depending on the activity of the efflux proteins in these cells and of the 11β-HSD2, that inactivate cortisol. In the aqueous humor, ∆4A was the only measured androgen, as testosterone was below quantification in all samples. Using Elisa assays, testosterone and progesterone were measured previously in the aqueous humor of patients undergoing cataract surgery^46^, and testosterone was measured at very low levels (around 0.1 ng/ml) in the vitreous of patients with various types of vitreoretinal diseases^47^. But it was shown that compared to LC–MS/MS, immunoassays considerably overestimate testosterone, androstenedione and 17-OHP levels in the serum^48^. It is now widely established that LC–MS/MS quantification is more accurate than immunoassays. The levels of the different corticoids measured in our experiments and particularly the cortisol level could have been influenced by the surgical stress and thus overestimated. But it was shown that before and during outpatient cataract surgery, surgical fear and stress is minimal and that although significantly higher fear scores could be measured occasionally in some types of patients, it was not correlated to biological parameters such as salivary cortisol or alpha-amylase^49^.

### Ocular steroidome does not correlates with systemic steroidome

In the serum, E/F and A/B levels significantly and positively correlated and F levels correlated with the time of the day, with higher levels being measured in the morning as compared to the evening as expected. Correlation of E levels with time was not significant although there was a tendency for higher E levels in the morning. F/E remained stable during the day as previously observed^50^.

In the aqueous humor, cortisol and cortisone levels were below 10% of serum levels indicating that active mechanisms might protect their entry into the eye and/or that only a fraction of free cortisol pass through the ocular barriers. Cortisol affinity to corticoid binding proteins is higher than cortisone affinity and cortisone has higher affinity for albumin^51^, but both corticoids showed similar ratio between the serum and the ocular media suggesting that mechanisms might control the passage of both bound and free forms. Indeed, cortisol and corticosterone were shown to be differential substrates for the multidrug resistance transporters, P-glycoprotein (P-gp, ABCB1) and ABCC1 (MRP1) that limit penetration of highly lipophilic molecules from the blood into the brain^52, 53^ and are expressed and active at the blood aqueous and blood retinal barriers, including in choroidal endothelial cells^54^. It can thus be hypothesized that like in the brain, efflux transporters control and regulate the entry of gluco and mineralocorticoids into the eye. But interestingly, although there was no correlation between serum and aqueous levels of cortisol and particularly no decrease of cortisol levels at the end of the day, cortisone serum level correlated with aqueous humor level which could indicate that cortisone could pass through the ocular barriers more easily than cortisol. In the rat, there was no difference in corticosterone levels in any brain areas of P-gp-deficient mice, but the brain uptake of cortisol was increased in the hypothalamus of P-gp-deficient mice, demonstrating the differential affinity of efflux transporters for the different corticoids in specific brain regions^55^. Similarly, testosterone, dihydroepiandrosterone (DHEA), androstenedione and dihydrotestosterone were shown to be P-gp ligands, explaining that they do not penetrate freely into the ocular media or the brain^56^.

The metabolism of ocular corticoids remains poorly understood. Whether the eye could produce locally cortisol and/or aldosterone has been debated. Although Zmijewski et al.^27^ showed cortisol could be produced from cholesterol in ARPE-19 cell line, we could not measure any cortisol produced from human RPE/choroid fresh organocultured tissues or from human RPE from iPS cells^25^. Most probably, cortisol in the eye comes from the circulation and its ocular entry is tightly controlled by its binding to proteins and/or by efflux proteins at the blood aqueous and blood retinal barriers.

From the present results, we hypothesize that cortisone could penetrate more easily from the blood into the eye and then could be transformed into the active form cortisol by the 11β-HSD1 enzyme offering a double control of the cortisol level in the eye, by its transfer into the eye on the one hand and by the enzymatic activity of HSD on the other. However, in different ocular cell types, we cannot exclude that different corticoids could be delivered in a very targeted manner. Other techniques should be used to explore the activity of efflux proteins towards different corticoids in the different ocular cells.

### Ocular steroidome is not influence by the age and time of the day

Interestingly, cortisol level in the eye seems to be highly controlled such as no circadian variation could be observed. On the other hand, we found that cortisone levels increase in the evening in the AH and not in the serum. Since F level did not decrease in the AH in the evening, the increase in E levels could rather result from a differential activity of the efflux proteins than from the activity of the 11-βHSD enzymes, which is supported by the absence of change in the B/A ratio with the time of the day. This hypothesis is supported by a recent observation that the circadian clock regulates efflux by the blood–brain barrier in mice and human cells, with a higher activity of the enzymes during the active phase^57^.

There was no influence of age in any of the corticoids measured in the aqueous humor, although one limitation of the study is that AH being collected during cataract surgery, steroidome was not performed in individuals younger than 29 years. There was no influence of the sex on the levels of F, E, A and B, but ∆4 levels were higher in men as compared to women, which can be explained by the fact that ∆4 is an androgen hormone produced both by the adrenal gland and by the testis. Finally, in a limited number of patients, for which we could perform a full steroidome in the AH and in the vitreous collected during the same surgical procedure, no difference was measured in the levels of E, F, A and B between compartments. A very good correlation between the levels of F in the AH and in the vitreous indicates that sampling of the AH could be relevant to evaluate F levels in the vitreous. We recognize that the number of subjects is low to draw a definite conclusion although similar results were previously observed when measuring cortisol in the aqueous and in the vitreous of different subjects^25^.

### Ocular steroidome in complex inactive CSCR eyes reveals imbalance towards reduced gluco and increased mineralocorticoids

Having shown that the ocular steroidome did not correlate with the systemic steroidome, we then analyzed the steroidome in the aqueous humor of a group of long lasting and complex CSCR, who did not show subretinal fluid at the time of sampling. We indeed chose a time when there was no obvious disruption of the blood-retinal barrier to avoid potential aggravation due to the surgery. On the other hand, the specific ocular steroidome of CSCR eyes could not have been interpreted in case systemic corticoid leakage had contaminated the ocular microenvironment, thus the levels we have measured cannot be attributed to the subretinal fluid. In the aqueous humor of CSCR patients, both cortisol and F/E ratio were significantly lower than in controls. After matching with samples collected at the same time of the day, the cortisone (E) levels and F/E ratio remained lower in CSCR as compared to controls indicating an imbalance in the gluco and mineralocorticoid pathways, which is supported by higher B/A ratio. This imbalance could result from a differential activity of HSD enzymes and/or from differential passage of corticoids from serum to the eye in CSCR patients. Although, the present results that ocular steroidome is not influenced by the age, we perform a sub analysis by comparing the ocular steroidome of CSCR as compared to age-matched controls and although the control group was smaller, similar results were observed.

The consequence of reduced F/E on the mineralocorticoid and glucocorticoid receptors (MR/GR) pathways in human eyes in unknown. In rats, we showed that reduction in ocular corticosterone levels (which is the only glucocorticoid in rodents) secondary to HPA negative axis feedback or secondary to chronic social stress, was associated with GR/MR pathway imbalance towards MR pathway overactivation in the RPE/choroid, as shown by the expression of MR downstream targets in the rat RPE/choroid complex^35^.

### Potential consequence of ocular steroidome imbalance in CSCR

In the ocular media, the stable and controlled level of cortisol, appears crucial to maintain ocular homeostasis since overactivation of the MR pathway has been shown to be pathogenic^24, 25, 28, 29, 58^. We and others have shown that MR pathway overactivation is associated with pachychoroid-like phenotype^29, 30^, choroid and epithelium inflammation, edema^28, 59, 60^ and with choroidal neovascularization^61^. Thus, the ligand imbalance measured in the CSCR eyes, indicates possible overactivation of MR pathway. Further studies analyzing MR and GR downstream molecular targets in eyes of CSCR patients are required to confirm our observation.

In this limited group of CSCR eyes, we did not observe any significant correlation between intraocular corticoids and the subfoveal choroidal thickness but this should be confirmed in a larger CSCR population. Previously, Karahan et al.^62^ did not find any correlation between systemic cortisol and subfoveal choroidal thickness in healthy individuals, but a complete steroidomic analysis at the time of measurement could be more informative.

### Restoration of ocular corticoid imbalance by the combination of mineralocorticoid receptor antagonism and topical glucocorticoids

To shift the MR/GR balance towards glucocorticoid pathway activation, we already proposed in 2019 to combine oral mineralocorticoid receptor antagonists (MRA) with glucocorticoids drops^63^. More recently, Amanda Wong et al.^64^ reported beneficial effects of this association.

On the basis of quantitative analysis of corticoids in the eyes of patients with CSCR, we suggest that restoration of physiological corticoid levels within the eye might benefit to these patients in order to counter-regulated excess of MR pathway activation. MRA prevents activation of MR pathway by its ligands but it might not activate the GR pathway. Topical dexamethasone sodium phosphate or hydrocortisone sodium phosphate (0.33%) can activate the GR as it does penetrate inside the aqueous humor and the ocular tissues and allows an increase in cortisol endogenous levels in normal individuals^65–67^. Moreover, we showed that in experimental uveitis, only very low dose of intraocular dexamethasone (100 nM) restored the normal glucocorticoid receptor expression, that was down-regulated by LPS injection^50^. Local low dose of glucocorticoids might thus not only restore the endogenous corticoid imbalance but also favor the GR expression. Whether oral MRA is needed in combination to prevent the potential binding of glucocorticoids to MR, or whether drops alone could be sufficient should be evaluated in randomized trials. How long these patients should remain under treatment is unknown but if abnormal corticoids metabolism if confirmed in CSCR eyes, independently from the occurrence of subretinal fluid, treatment could be required on the long term. Whether other routes of administration or formulations should be preferred is under investigation.

### Limitations of the study

We recognize limitations of this study, such as the limited number of CSCR AH, but CSCR is a rare disease that affects mostly young individuals, explaining the rarity of those samples. Another limitation is that the steroidome of these patients has been measured when no subretinal fluid was present. It can therefore not be excluded that upon disruption of the outer retinal barrier, responsible for subretinal fluid, the steroidome could be different, which is currently under investigation. On the other hand, the fact that we observed abnormal metabolism of ocular corticoids in the inactive form of the disease demonstrates that it is not a consequence of the transient disrupted retinal pigment epithelium barrier. Other limitations include the design of the quantification method we have used, that chose to measure all corticoids (bound and unbound) in the different compartments. This choice was motivated by the fact that the different corticoids have different binding affinities for cortisol binding globulin, albumin and other corticoid binding proteins, that it is not known if blood retinal barrier can only be crossed by unbound forms or if protein-bound corticoid could also be transported at the blood ocular barriers and by the fact that no CBG was found in the human aqueous humor^23^. We believe that the use of highly sensitive quantification methods adapted to ocular environments opens the way to a better understanding of ocular corticoid metabolism LCMS/MS is the most accurate method to quantify the steroidome. However, further studies should include the quantification of CGB and albumin to get a better understanding on the passage of the different corticoids from the blood to the ocular compartments.

## Conclusion

In conclusion, this study shows a local metabolism of corticoids in the aqueous humor; independent from the systemic steroidome. Ocular levels of cortisol are not influence by the systemic cortisol levels including its nychthemeral variations. Corticoid levels in the aqueous humor does not seem to be influenced by age and sex. In eyes with long-lasting and complex but inactive CSCR, we observed an imbalance towards lower active glucocorticoids and higher active mineralocorticoid indicating that topical glucocorticoids might be beneficial for these patients. Further analysis in larger cohorts are needed to confirm these preliminary results.

## Data Availability

The datasets used and/or analysed during the current study available from the corresponding author on reasonable request.
